# Anthracycline antibiotics derivate mitoxantrone—Destructive sorption and photocatalytic degradation

**DOI:** 10.1371/journal.pone.0193116

**Published:** 2018-03-13

**Authors:** Irena R. Štenglová-Netíková, Luboš Petruželka, Martin Šťastný, Václav Štengl

**Affiliations:** 1 Department of Oncology, 1^st^ Faculty of Medicine, Charles University in Prague, Czech Republic; 2 Department of Material Chemistry, Institute of Inorganic Chemistry ASCR, Husinec-Rez, Czech Republic; 3 Faculty of Environment, J.E.Purkyně University in Ústí nad Labem, Czech Republic; VIT University, INDIA

## Abstract

Nanostructured titanium(IV) oxide was used for the destructive adsorption and photocatalytic degradation of mitoxantrone (MTX), a cytostatic drug from the group of anthracycline antibiotics. During adsorption on a titania dioxide surface, four degradation products of MTX, mitoxantrone dicarboxylic acid, 1,4-dihydroxy-5-((2-((2-hydroxyethyl)amino)ethyl)amino)-8-((2-(methylamino)ethyl)amino)anthracene-9,10-dione, 1,4-dihydroxy-5,8-diiminoanthracene-9,10(5H,8H)-dione and 1,4-dihydroxy-5-imino-8-(methyleneamino)anthracene-9,10(5H,8H)-dione, were identified. In the case of photocatalytic degradation, only one degradation product after 15 min at m/z 472 was identified. This degradation product corresponded to mitoxantrone dicarboxylic acid, and complete mineralization was attained in one hour. Destructive adsorbent manganese(IV) oxide, MnO_2_, was used only for the destructive adsorption of MTX. Destructive adsorption occurred only for one degradation product, mitoxantrone dicarboxylic acid, against anatase TiO_2_.

## 1. Introduction

Xenobiotics are foreign substances to the organism that enter the organism intentionally or involuntarily, and their undesirable or toxic effects are eliminated by various mechanisms. These foreign substances include industrially produced chemical compounds such as pharmaceuticals, preservatives and stabilizers in food, industrial chemicals, cosmetic ingredients, fertilizers, fungicides, herbicides, insecticides, detergents, etc. Polar or hydrophilic xenobiotics are rapidly excreted, while lipophilic compounds remain in the living organism and must be converted to more polar metabolites than the starting material before excretion. Many xenobiotics are rapidly degraded in the environment, but their transformation products are equally or even more toxic than the parent compound.

Cytotoxic agents are substances that are used to treat cancer, and their effect is to stop the growth of tumour cells. This effect is nonspecific, so it affects healthy cells as well. Cytostatic compounds are formed by a group of environmental contaminants whose presence, effects and risk are thus far unknown. Mitoxantrone is an anthraquinone that stops tumour growth by directly attacking DNA by cross-linking guanine bases in double helix chains of DNA. Mitoxantrone is excreted from the body in the urine at 6–11%, 25% in the faeces and the remainder in the metabolized form [1]. As with other medicinal products, parent products or metabolites are excreted and discharged into sewage through urban or hospital effluent and sewage treatment plants, where the effluents may be released into surface water. Because of their non-specific toxicity, cytotoxic agents can affect aquatic organisms.

Commonly used sorbents, such as activated carbon, zeolites, diatomaceous earth or kieselguhr, hazardous and toxic substances, adsorb only on their surface but do not decompose. Nanostructured light metal oxides (Ca, Mg, Al) of the adsorbed compound decompose into non-toxic decomposition products, preferably water, carbon dioxide and the corresponding mineral acid. Therefore, we use the name destructive adsorbents and the active adsorbent process for these nanostructured materials. Destructive sorbents based on Ca [2], Mg [3], [4] Al [5] and especially Ti [6] were used for stoichiometric degradation of warfare agents, pesticides or their stimulants. Because of the chemical nature of these substances, their degradation reactions always occur in a non-aqueous and non-polar environment, most often in hexane or higher aliphatic solvents. Ca, Mg and Al oxides were prepared for these degradation processes in a non-aqueous medium by high-temperature supercritical drying. Anatase, a crystalline modification of titanium dioxide, is in a nano-crystalline form an excellent photocatalyst as well as a destructive sorbent, depending on the reaction conditions of its synthesis. The natural properties of titanium dioxide, according to the preparation conditions, allow degradation reactions to occur on its surface in water and polar solvents.

Mustine, (HN_2_, bis(2-chloroethyl)methylamine, (C1CH_2_CH_2_)_2_NH)) is derived from sulfur mustard (HD, bis(2-chloroethyl) sulfide, (C1CH_2_CH_2_)_2_S)) by nitrogen substitution for sulfur and is a prototype alkylating agent used as a chemotherapeutic. The family of nitrogen mustard also contains HN1 bis(2-chloroethyl)ethylamine and HN3 tris(2-chloroethyl)amine, classified as warfare agents, and cyclophosphamide, ifosfamide, chlorambucil, uramustine, melphalan and bendamustine, which are used as cytostatic agents. Štenglová Netíková *et al*. [7] presented the stoichiometric degradation of cytostatics from nitrogen mustards (cyclophosphamide and ifosfamide) and anthracycline antibiotics (doxorubicin and epirubicin) to nanocrystalline anatase.

In this publication, therefore, we attempt to determine the decomposition products of the mitoxantrone reaction with sodium hydroxide and sodium hypochlorite solution, which simulate common cleaning agents used in healthcare facilities. For comparison, the reaction with nanocrystalline titanium and manganese oxides as destructive adsorbents was also performed.

## 2. Experimental

All chemicals used, titanium oxo-sulfate (TiOSO_4_), urea (CO(NH_2_)_2_), potassium permanganate (KMnO_4_), 2-chloroacetamide (CH_2_CONH_2_Cl), sulfuric acid (H_2_SO_4_), hydrochloric acid (HCl), sodium hydroxide (NaOH), and sodium hypochlorite (NaClO) were obtained from Sigma-Aldrich, and mitoxantrone was used as drugs from EBEWE Pharma.

### 2.1. Titanium(IV) oxide TiO_2_, anatase modification synthesis

The method for the homogeneous hydrolysis of titanium oxo-sulfate with urea for nano-structured titanium(IV) oxide was used [6], [7, 8]. In a typical synthesis, 30 g of TiOSO_4_ was dissolved in 100 mL of hot distilled water acidified with 98% H_2_SO_4_. The pellucid liquid was diluted into 4 L of distilled water, and 300 g of urea was added. The mixture was heated at 98°C under stirring for 6 h until the pH reached 7.2. The formed precipitate was washed using decantation until a conductivity of 10 μS/cm was reached, filtered, and dried at 105°C.

### 2.2. Manganese(IV) oxide δ/MnO2, birnessite modification synthesis

Manganese(IV) oxide was prepared by homogeneous hydrolysis of potassium permanganate with 2-chloroacetamide [9] [10]. In a typical experiment, 15.8 g of KMnO4 was dissolved in 4 L of distilled water, and 37 g of 2-chloroacetamide was added. The reaction mixture was adjusted to pH 2 with hydrochloric acid (HCl). The reaction mixture was heated at ±100°C and stirred for 6 h. Then, the synthesized sample was washed with distilled water by decantation, filtered, and dried at 105°C in an oven.

### 2.3. Characterization of methods

The diffraction pattern was collected with a Bruker D2 diffractometer equipped with a conventional X-ray tube (Cu Ka radiation, 30 kV, 10 mA) and an LYNXEYE 1-dimensional detector. A primary divergence slit module width of 0.6 mm, a Soler Module 2.5, an Airscatter screen module of 2 mm, a Ni Kbeta-filter of 0.5 mm, a step of 0.00607°, and a time per step of 0.3 s were used. Qualitative analysis was performed with the DiffracPlus Eva software package (Bruker AXS, Germany) using the PDF-2 database.

The surface area of the samples was determined from the nitrogen adsorption-desorption isotherm at liquid nitrogen temperature using a Coulter SA3100 instrument with 15 min of outgassing at 150°C. The Brunauer–Emmett–Teller (BET) method was used for surface area calculations. Scanning electron microscopy (SEM) analysis was conducted on an FEI Nova NanoSEM scanning electron microscope equipped with an Everhart–Thornley detector (ETD) and through-lens detector (TLD) at an accelerating voltage of 4–30 kV. The samples were deposited on the carbon holder. The morphology of the prepared samples was inspected by high-resolution transmission electron microscopy (HRTEM) using a 200-kV HRTEM microscope (FEI Talos F200X), which combines high-resolution S/TEM with energy dispersive X-ray spectroscopy (EDS) signal detection and 3D chemical characterization with compositional mapping. As a specimen support for TEM investigations, a microscopic copper grid covered by a thin transparent carbon film was used.

The visible spectra at 400–800 nm of MTX were determined with a UV/VIS spectrophotometer ColorQuest with an Ulbricht sphere.

#### 2.3.1. Destructive adsorption / stoichiometric degradation—HPLC/MS

All samples were taken after degradation to analyse potential degradation products using an HPLC/MS method. Mitoxantrone analysis was performed using an LTQ Orbitrap mass spectrometer following chromatographic separation using a liquid chromatography (HPLC) system consisting of an autosampler and dual pumps. A Thermo Finnigan HPLC system (Palo Alto, CA, USA) was used. The chromatographic separations were carried out by automated injection of 10 μL samples onto a Kinetex column 3 mm × 100 mm packed with an octadecyl-bonded stationary phase (Gemini, C-18, 5 μm; Phenomenex, Torrance, CA, USA), with a gradient mobile phase adopted: 30/70–90/10 methanol/water in 40 min in ESI positive mode at a flow rate of 1 mL/min. An LTQ Orbitrap mass spectrometer (Thermo Scientific, Bremen, Germany) equipped with an atmospheric pressure interface and an ESI ion source was used. The tuning parameters adopted for the ESI source were as follows: capillary voltage, 37.00 V and tube lens, 65 V. The source voltage was set to 3.5 kV. The heated capillary temperature was maintained at 275°C. Analyses were run using full-scan MS (50–1000 m/z range) in positive ion mode, with a resolution of 30.000 in ITMS mode.

Decontamination tests were performed using MTX as an internal standard (2 mg/mL). In this procedure, the standard was mixed into the decontamination solution in a series of glass vials (Supelco, 20 mL). The vials were sealed with caps and covered with aluminium foil to protect the reaction mixture from sunlight. At predetermined time intervals (0, 5, 15, 20, 30, 50, 70, 90, and 120 min), the reaction was terminated by the addition of formic acid (0.1%). The solution was transferred to a smaller vial (Supelco, 2 mL) and analysed immediately by HPLC-MS to determine oxidation or hydrolysis transformation products (TPs).

#### 2.3.2. Destructive adsorption / stoichiometric degradation—FTIR

The surface interaction between titanium oxide and drug molecules was studied on an FTIR spectrometer (Nicolet Impact 400D) equipped with a Praying MantisTM (Harrick) for diffuse reflection measurement (DRIFTS). Heating of the cell was controlled by ATC-024-3 equipment (Harrick). One drop of mitoxantrone was dosed onto the oxide surface using a syringe.

#### 2.3.3. Photocatalytic degradation and kinetics of destructive adsorption

The photocatalytic degradation and kinetics of the sorption of mitoxantrone on surface titanium oxide were determined in a self-constructed photoreactor [11], [12]. It consisted of a stainless steel cover and an inner quartz tube with a fluorescent lamp (Narva, Black Light, 365 nm) with a power of 13 W producing a light intensity of ∼3.5 mW/cm2; the emission spectrum is shown in [13].

The 5mL stock solution of mitoxantrone at a concentration of 2 mg/ml was diluted to a total volume of 1 L and circulated using a membrane pump through a flow cell. Mitoxantrone has a blue colour, with two absorption maxima at 600 and 650 nm. The concentration was determined by measuring the absorbance at 650 nm with a visible spectrophotometer (ColorQuest XE). A portion of 0.5 g of photocatalytic titanium(IV) dioxide was dispersed in an ultrasonic bath (300 W, 35 kHz) for 10 min before the photocatalytic or kinetic tests; the method of dispersing the oxide plays a crucial role in obtaining reproducible results from both tests. The kinetic experiment started by switching on the light source after the spectral signal of mitoxantrone in the suspension reached steady state; this initial signal was taken as a measure of the initial concentration of mitoxantrone.

The time dependence of mitoxantrone photocatalytic degradation (and kinetics of sorption on the titania surface) was fitted using Eq (1); alternatively, Eq (2) was modified for the first-order kinetics reaction [14]:

$$-dc/dt=k(c_{0}-c)$$ (1)

$$-dc/dt=k_{1}(c_{0}-c)+k_{2}(c_{0}-c)+A$$ (2)

where c is the concentration of mitoxantrone; c_0_ is the initial concentration of mitoxantrone; and k, k_1_, and k_2_ are the rate constants. A is a constant expressing the unreacted proportion of mitoxantrone.

The adsorption and photodegradation in the mechanism of degradation of MTX in water in the presence of titania was assessed at predetermined time intervals (0, 5, 15, 30, 50, 70, 90, and 120 min), the sorbent was immediately separated by centrifugation (15000 rpm for 4 min) and the supernatant was transferred to a glass vial (Supelco, 4 mL) and analysed immediately by a UV/VIS spectrophotometer ColorQuest XE was used to determine the possible change in the absorption maximum. The HPLC-MS method was used for the quantification of MTX removal and identification of degradation products (DPs), and LC-MS was used to determine the hydrolysis and eventual formation of DPs. Mitoxantrone and DPs were unequivocally identified by their already described UV–VIS spectral features [15] and their parent molecular ion mass [M+H]^+^ at m/z 445 [16]. Mitoxantrone has a UV–VIS spectrum with maximum absorbance at 590 and 651 nm, and its parent molecular ion mass [M+H]^+^ is m/z 445.

## 3. Results and discussion

The XRD pattern of the titanium(IV) oxide sample prepared by homogeneous hydrolysis of titanium oxo-sulfate (TiOSO_4_) with urea is presented in Supplement “Fig S1”. Single-phase anatase was obtained, and an interlayer spacing of d_101_ = 0.3504 nm corresponds to card ICDD PDF 21–1272. The broadening of diffraction peaks indicates a small size of the titania nanocrystals. The average size *t* of crystallites was calculated from the full width at half maximum (FWHM) using the Scherrer equation [17],

t=k⋅λ/B∙cos(Θ) (3)

where *k* is a shape factor of the particle (it is equal to one if the spherical shape is assumed) and *λ* and *θ* are the wavelengths and the incident angle of the X-rays, respectively. The crystallite size *t* = 8.4 nm was calculated from a diffraction plane (101) and corresponded to the HRTEM investigation. Supplement “Fig S2a and b” shows SEM images, and Supplement “Fig S2c” shows TEM images of the prepared TiO_2_ sample as spherical particles. Crystallite areas of ~4 nm in size can be observed in the HRTEM image of TiO_2_ (see Supplement “Fig S3”). The interlayer spacing is calculated from the electron diffraction (ED) inset in Supplement “Fig S3”. The interlayer spacing for line(1) d = 0.347 nm corresponds to d_101_ = 0.352 nm, line(2) d = 0.239 nm to d_004_ = 0.238 nm, line(3) d = 0.206 nm to d_200_ = 0.189 nm, line(4) d = 0.171 nm to d_105_ = 0.169 nm and line(5) d = 0.150 nm to d_421_ = 0.149 nm. These results confirm the high crystallinity of the prepared anatase TiO_2_ sample.

Supplement “Fig S4a” gives the nitrogen adsorption isotherm, and Supplement “Fig S4b” gives the pore volume distribution of the synthesized TiO_2_ sample. The isotherm shows a typical IUPAC type IV pattern of a type H2 hysteresis loop, indicating the existence of mesopores 4–5 nm in size. The BET surface area and total pore volume of the TiO_2_ sample were determined to be 318.9 m^2^/g and 0.2347 cm^3^/g, respectively.

**Fig 1:**
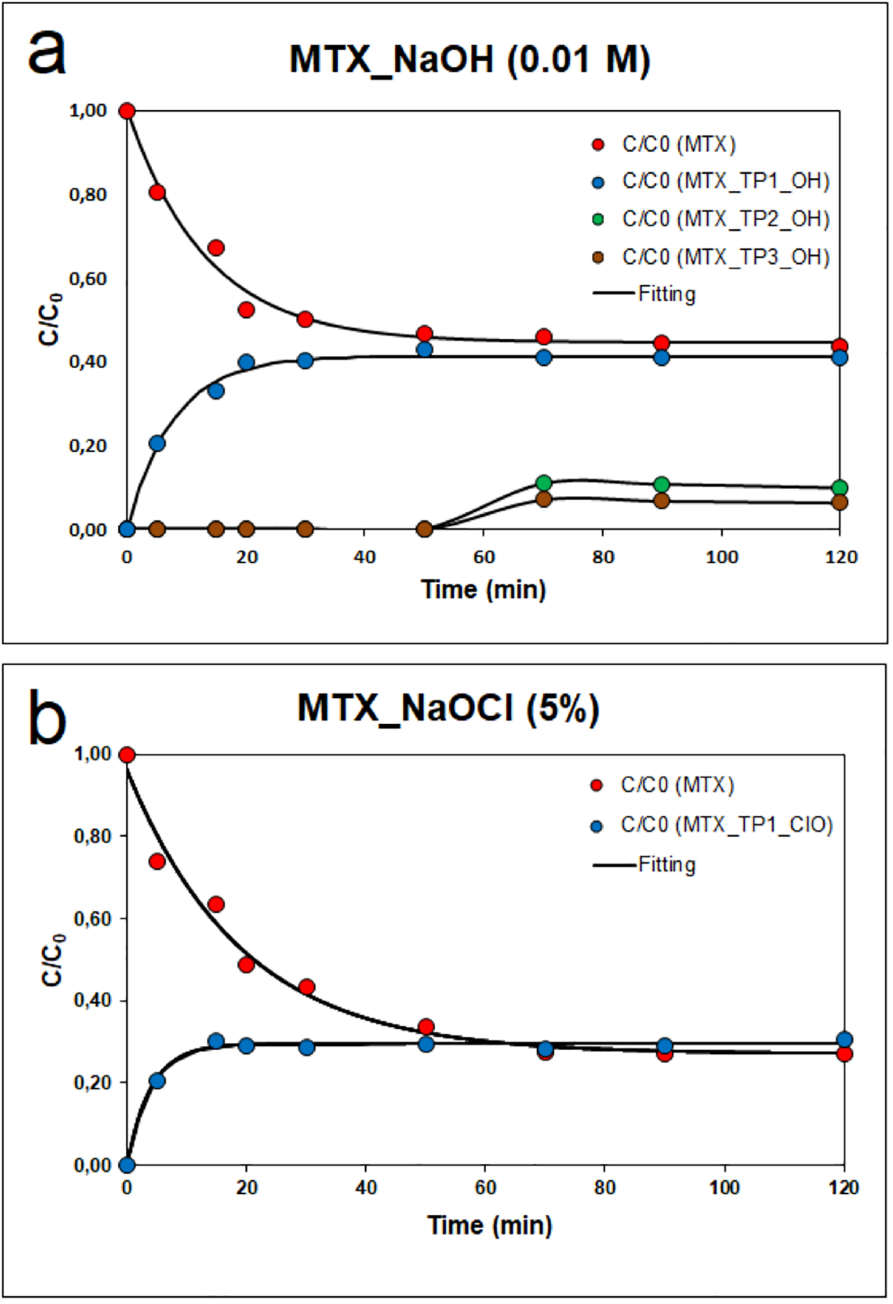
Kinetics of the degradation of mitoxantrone in the presence of a) 0.01 M NaOH and b) 5% NaOCl.

Supplement “Fig S5” shows the HPLC–MS chromatograms and mass spectra of mitoxantrone (MTX). Transformation products detected during the reaction of MTX with sodium hydroxide (NaOH) in ESI positive mode are shown in Supplement “Fig S6”, where mitoxantrone at 2.70 min is present. At 5 and 60 min, three transformation products (TPs) of MTX were identified. The degradation kinetics of MTX in NaOH is presented in “Fig 1a”; the calculated rate constant is k = 0.0754 min^-1^ and the corresponding degree of conversion MTX is 58.2%.

**Fig 2:**
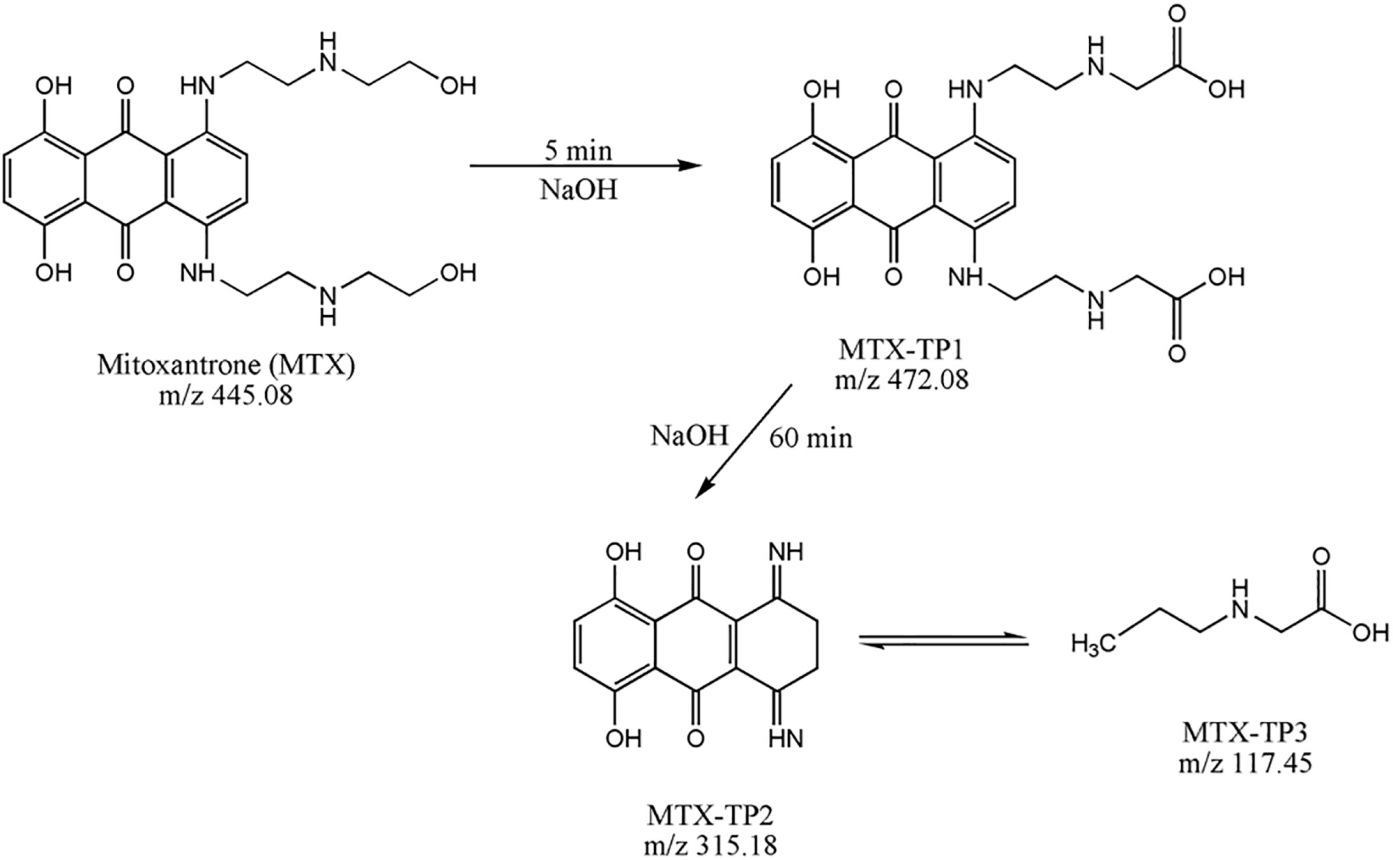
The proposed pathway of the conversion of mitoxantrone (MTX) in the presence of 0.01 M sodium hydroxide.

Mitoxantrone in 0.01 M NaOH solution reacts to form three transformation products, and the proposed pathway of conversion is presented in “Fig 2”. First, labelled MTX-TP1-OH was identified at 5 min at m/z 472.08 (t_R_ = 4.74 min), corresponding to mitoxantrone dicarboxylic acid (MTOD) generated by oxidation of the terminal hydroxyl groups of the side chains of MTX. After 60 min, next two reaction products were identified: 1,4-dihydroxy-5,8-diiminoanthracene-9,10(5H,8H)-dione at m/z 315.18 (t_R_ = 4.57 min) labelled MTX-TP2-OH, corresponds to the loss of the two amino alcohols (N-ethyl ethanolamine) from the original molecule as the fragment [C_4_H_10_NO^+^], which was labelled MTX-TP3-OH (m/z 117.45; t_R_ = 5.23 min). This breaking of the aliphatic chains produces a relocation of the non-bonded electron pair of nitrogen, which makes an electronic rotation to convey stability to the structure. This effect can be observed in mitoxantrone’s modified preparation by De Leoz *et al*. [18], who reported the preparation of MTX from the intermediate leuco-tetrahydroxyanthraquinone. The reaction involves condensation of the leuco-tetrahydroxyanthraquinone intermediate with an amino alcohol (2-(2-aminoethyl amino)ethanol) to form a Schiff base, which is oxidized to the final product mitoxantrone with the use of either dry or wet air. After 60 minutes, no other transformation products have been identified, and MTX-TP2-OH and MTX-TP3-OH remain undecomposed in the reaction mixture.

**Fig 3:**
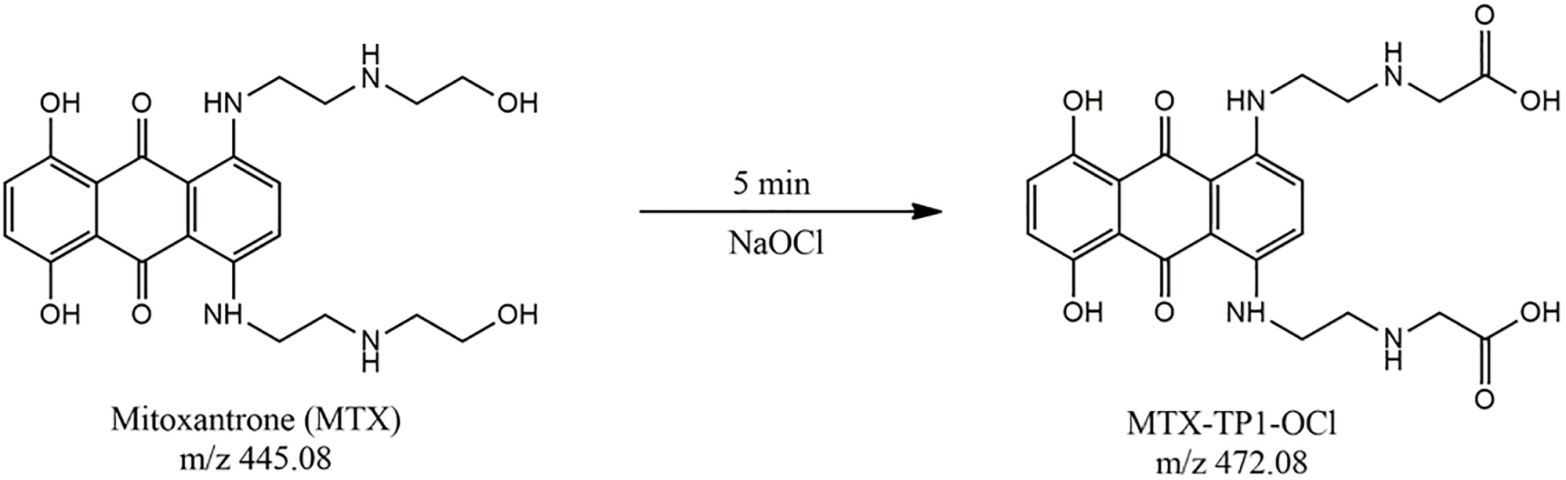
The proposed pathway of the conversion of mitoxantrone (MTX) in the presence of 5% sodium hypochlorite.

In the case of transformation of mitoxantrone in the presence of 5% solution sodium hypochlorite (NaOCl) as a decontamination agent, only one oxidation product labelled MTX-TP1-ClO after 5 min at m/z 472.08 (t_R_ = 4.73 min) was identified, corresponding to mitoxantrone dicarboxylic acid (MTOD), as shown in Supplement “Fig S7”. The calculated rate constant was k = 0.0521 min^-^1, and the degree of conversion MTX was 72.8% (see “Fig 1b”) [8]. The proposed pathway of conversion of MTX in the presence of 5% sodium hypochlorite agent is presented in “Fig 3”.

As has been found, mitoxantrone, similar to doxorubicin and epirubicin [7], has a high affinity for a TiO_2_ surface prepared by homogeneous hydrolysis. For example, a 1 mL solution of mitoxantrone at a concentration of 2 mg/mL was adsorbed to the surface of 0.25 g TiO_2_ within 5 min (see Supplement “Fig S8”).

Significant degradation pathways of MTX, as well as its degradation (transformation) products, were detected in the solution (the TiO_2_ (MnO_2_)-water-MTX system) by an HPLC-MS method, which was validated for these purposes [7]. The surface reaction (the MTX-water-TiO_2_ system) was monitored by DRIFT spectra detected in situ over the reaction time. DRIFT spectral data provide the most definitive means of identifying the surface species generated upon destructive adsorption and the corresponding chemical interactions of MTX (fragments) with titania surfaces [19].

The results of the destructive adsorption experiments in the heterogeneous MTX-water-TiO_2_ (MnO_2_) system indicated the formation of degradation products that were extracted from the titanium (manganese) surface into the solution and were immediately measured in the supernatant by the HPLC-MS method. Rapid adsorption of MTX occurred in the presence of TiO_2_ or MnO_2_ in water, and this compound disappeared completely after 120 min.

**Fig 4:**
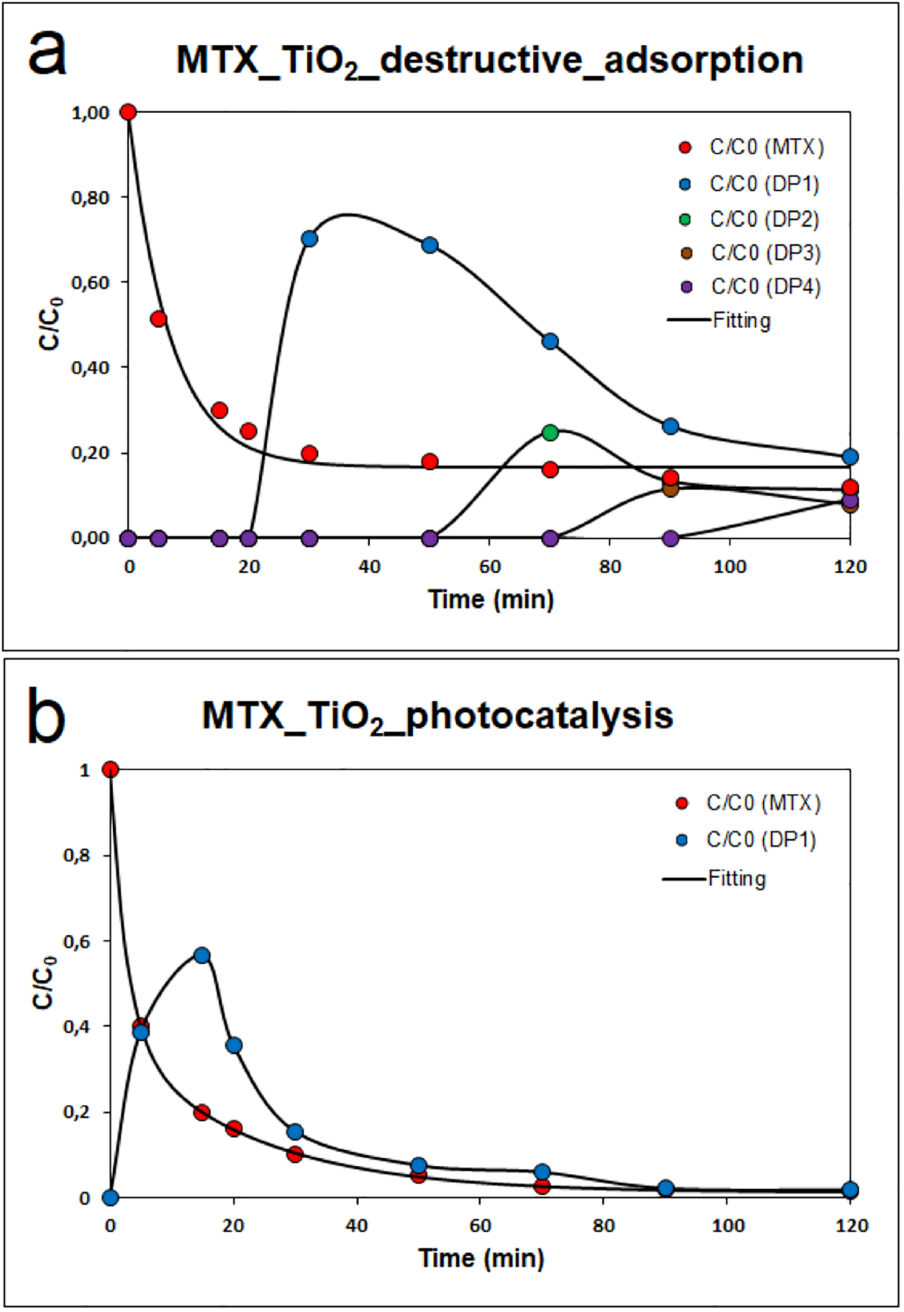
Kinetics of mitoxantrone degradation in the presence of TiO_2_ a) sorption and b) photocatalysis.

Supplement “Fig S9” shows the HPLC–MS chromatograms and mass spectral detection during destructive adsorption of mitoxantrone in the presence of titanium oxide in ESI positive mode, where the trace of mitoxantrone at 2.73 min is present (see Supplement “Fig S5”). At 30, 60, 90 and 120 min, four degradation products were identified. The kinetics of mitoxantrone sorption and the balance of the growth of degradation products on the titania surface are visible in “Fig 4a”. The rate constant of the first-order calculation was determined as k = 0.1435 min^-1^, and the degree of conversion at 120 min was 89.9%.

**Fig 5:**
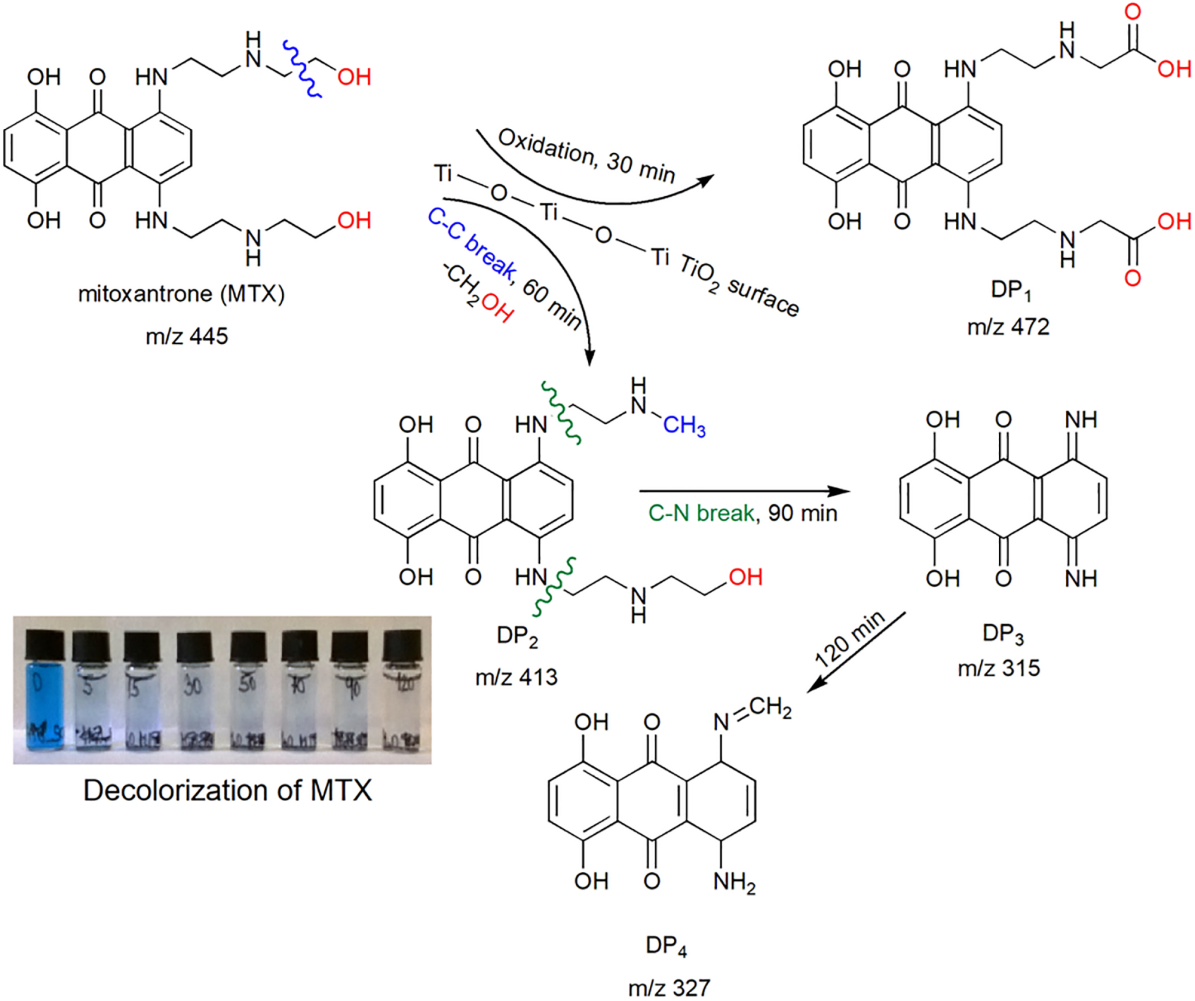
Schematic of the sorption of mitoxantrone on a titania surface and the formation of degradation products.

The first degradation product (DP1) was identified after 30 min at m/z 472 (t_R_ = 4.76 min), which corresponded to mitoxantrone dicarboxylic acid (MTOD) generated by oxidation of the terminal hydroxyl groups of the side chains of MTX [20]. This reaction step is the same as for the reaction with NaOH and NaOCl. At 60 min, the second degradation product (DP2) was identified at m/z 413 (t_R_ = 3.78 min) and corresponded to the loss of CH_2_OH from the C-C bond breakage in the original structure [21]. A third degradation product (DP3) of the molecule MTX at m/z 315 (t_R_ = 4.51 min) min was identified at 90 min as 1,4-dihydroxy-5,8-diiminoanthracene-9,10(5H,8H)-dione and corresponded to the loss of *N*-ethylmethylamine (C_2_H_5_NHCH_3_) and 2-(ethylamino)ethanol (C_2_H_5_NHCH_2_CH_2_OH) from the C-N bond breakage in the second degradation product (DP2). Neither derivative was identified in the solution [22]. DP3 is the same degradation product labelled MTX-TP2-OH in the MTX reaction with NaOH. At 120 minutes, the fourth degradation product was identified at m/z 327 (t_R_ = 3.25 min) as 1,4-dihydroxy-5-imino-8-(methyleneamino)anthracene-9,10(5H,8H)-dione. These degradation products are not stable, and after 120 min, no degradation products were identified. A schematic of the sorption of mitoxantrone on the titania surface and the formation of degradation products is shown in “Fig 5”. The degrees of conversion for the degradation products were DP1 = 82.1%, DP2 = 89.0%, DP3 = 92.1% and DP4 = 90.9%.

Mitoxantrone was unequivocally identified on the basis of its already described VIS spectral features (see Supplement “Fig S10”) and has a VIS spectrum with maximum absorbance at 590 and 651 nm [15]. Its parent molecular ion mass [M+H]^+^ was m/z 445 (see Supplement “Fig S5”). Supplement “Fig S10a” shows the VIS spectra of MTX and its degradation products scanned during sorption on the titania surface. The degradation product DP2 presents a maximum absorbance peak at 561 nm. Degradation product DP1 and degradation product DP3 present a VIS spectrum similar to mitoxantrone, and degradation product DP4 was not visible in the VIS spectrum. These four DPs were not stable during the 150 min of the experiment. After 120 min, no degradation products were identified. The detailed mechanism of mitoxantrone adsorption on the surface of titanium oxide is shown in “Fig 5”.

The use of TiO_2_ also makes it possible to degrade mitoxantrone because the electrons can potentially oxidize it in the conduction band [23]. Mitoxantrone shows weak photolysis and spontaneous decomposition under UV light [1]. For the formal kinetic description of heterogeneous photocatalysis, the Langmuir–Hinshelwood equation [24] can be used, whereas mitoxantrone has a similar absorbance spectrum in the visible region, such as some azo-dyes, e.g., Reactive Black 5 [25]. In the case of mitoxantrone photocatalytic degradation in the presence of titanium oxide as a heterogeneous catalyst, only one degradation product (DP1) was identified after 15 min at m/z 472, corresponding to mitoxantrone dicarboxylic acid (MTOD), as shown in Supplement “Fig S11a”.

**Fig 6:**
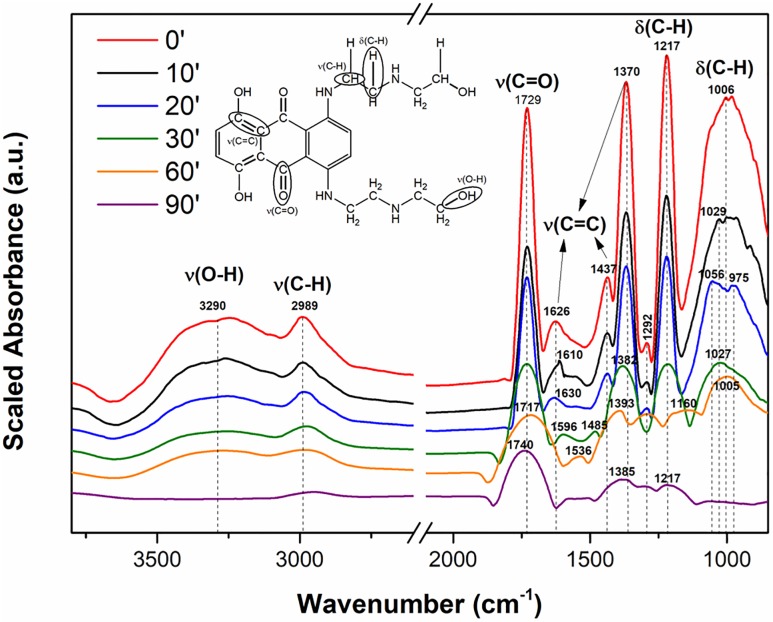
Schematic of the photodegradation of mitoxantrone (MTX) and the formation of degradation product 1 (DP1) on a titania surface.

Supplement “Fig S10b” shows the efficiency of titanium oxide for the photocatalytic degradation of mitoxantrone shown by spectrophotometric measurements. The kinetic curve of the photocatalytic degradation of MTX is shown in “Fig 4b”. The rate constants k calculated in two first-order calculations [26] were determined as k_1_ = 0.3717 min^-1^ and k_2_ = 0.0462 min^-1^. The constant k_1_ expresses the initial, faster and dominant part of the reaction kinetics, whereas k_2_ serves to express the slower and final part of the reaction. Titanium oxide was the most efficient for photocatalytic mineralization of mitoxantrone, with 98% total organic carbon removal. A schematic of the photodegradation of mitoxantrone (MTX) and the formation of degradation product 1 (DP1) on the titania surface is shown in “Fig 6”.

**Fig 7:**
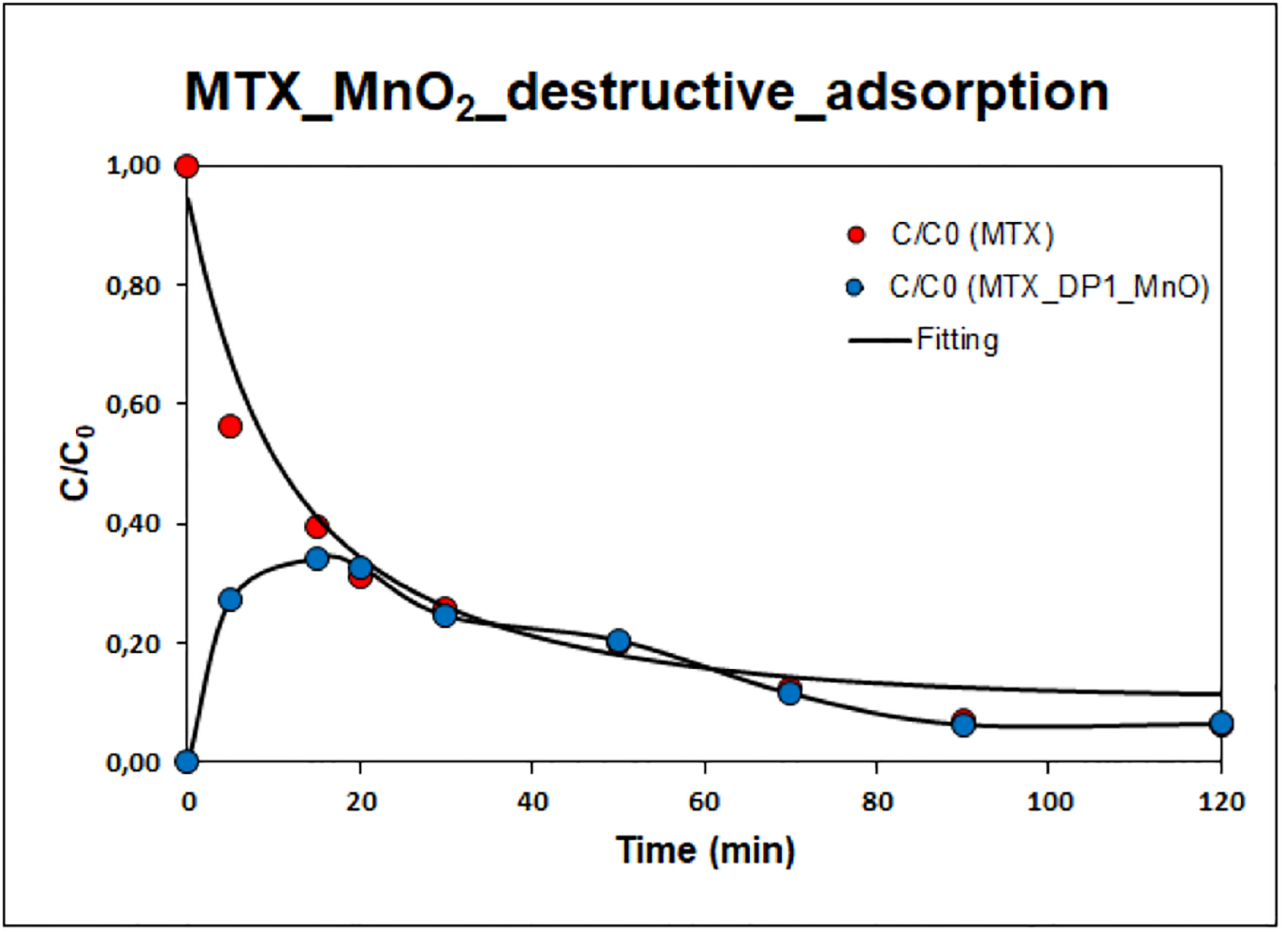
Kinetics of mitoxantrone degradation in the presence of MnO_2_.

As the next most destructive adsorbent for mitoxantrone degradation, birnessite-type manganese (IV) oxide was used. The kinetics of MTX degradation are shown in “Fig 7”, the calculated rate constant k_1_ = 0.1332 min^-1^ corresponded to the faster beginning of the adsorption process, and k_2_ = 0.0352 min^-1^ corresponded to the slower end of the degradation process. Destructive adsorption, unlike titanium oxide, occurs only through one intermediate product, namely, mitoxantrone dicarboxylic acid. Supplement “Fig S12” shows the HPLC-MS chromatogram and mass spectra of the transformation products of mitoxantrone on manganese(IV) oxide. The degree of conversion MTX after 120 min was ~ 93.6%.

Compared to the degradation of doxorubicin and epirubicin [7] on the surface of titanium oxide, the photocatalytic degradation of mitoxantrone, even though it occurs via several intermediates, is much faster. In contrast, the stoichiometric decomposition of doxorubicin and epirubicin on the TiO_2_ surface is faster than that of mitoxantrone. After 5 minutes, only daunosamine was detected in the solution and remained in the solution.

**Fig 8:**
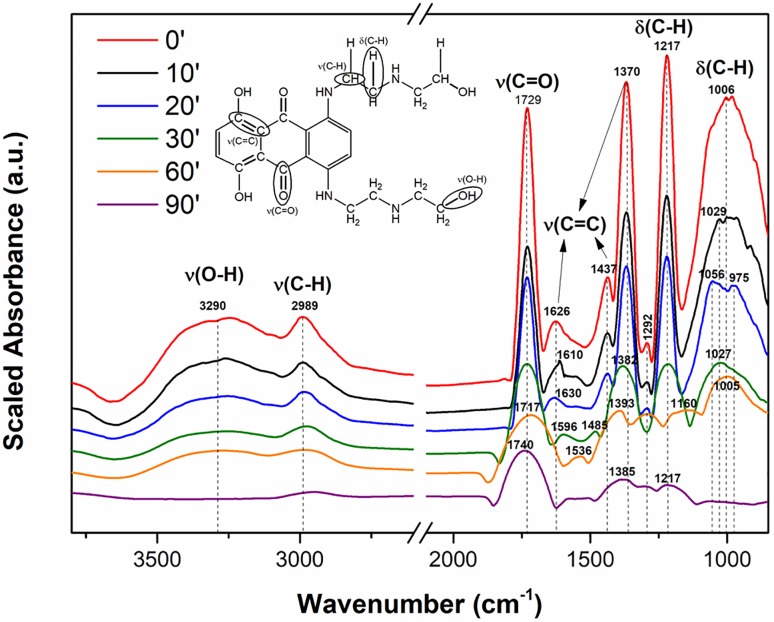
Chemical structure of MTX with labelled bonds and DRIFT spectra of MTX (red curve) and its adsorption on a TiO_2_ surface over the reaction time.

However, from the DRIFT spectra obtained during the reaction time for the MTX-TiO_2_ system, some important bands from the surface-adsorbed MTX are recognizable. To further demonstrate the successful destruction of MTX, DRIFT spectra were evaluated to compare the peaks of pure MTX (see red curve in “Fig 8”).

The DRIFT spectra in “Fig 8” show several important peaks: In the range of approximately 1400 to 1620 cm^-1^ is evident a ring mode peak that involves the stretching and contracting of the carbon-carbon (C = C) bonds in aromatic rings. The peaks of the pure drug molecules are sharp, but during destructive adsorption, these bands are downshifted from 1626 to 1536 cm^-1^ (and 1437 to 1485 cm^-1^) to give wider peaks that cannot be uniquely identified due to the resolution of the instrument [27]. The most intense peaks in “Fig 8” corresponding to wavenumbers 1006 and 1217 cm^-1^ were assigned to out-of-plane C-H bends in side-chain alkyl groups. During the adsorption process over time, these belts shifted to lower wavelengths (approximately 1005 and 1160 cm^-1^ due to decomposition of these bonds [28]. Additionally, the slight peak appearing at 1292 cm^−1^ represents the amide III bands with lower absorption intensity (decreased after 60 minutes) [29].

The narrow stretching vibration band of ν(C = O) at 1729 cm^−1^ originated from carbonyl groups on the middle hexagon of the MTX molecule. Even in this case, the shifting of the vibration mode to higher wavelengths (approximately 1740 cm^-1^) and the simultaneous diffusion of the individual bands were evident [30]. Scrutiny of the DRIFT spectra at 2989 and 3290 cm^-1^ shows that these bands completely disappeared due to the degradation of the mitoxantrone molecule. These two bands were assigned as ν(O-H) and ν(C-H) bending, respectively. The O-H vibration comes from the surface-bound (Ti-OH) groups on titanium oxide, and their disappearance was related to the participation of these groups in the degradation process [31].

Changes in the intensity and shifting of vibration modes can indicate destruction of the MTX molecule on the TiO_2_ surface. For manganese oxide, the DRIFT spectra were not measured due to the high absorption of infrared radiation by the sample.

## 4. Conclusions

Destructive adsorbents based on nanostructured metal oxides, anatase TiO_2_, and birnessite δ-MnO_2_ were used to degrade mitoxantrone. The results were compared with simulated decontaminants currently in use, 0.01 M NaOH and 5% NaOCl. It can be clearly stated that decontamination using conventional detergents is insufficient to degrade mitoxantrone, resulting in only partial degradation and producing intermediates that do not decompose even after 120 minutes. Titanium(IV) oxide and manganese(IV) oxide are able to degrade mitoxantrone on their surface without producing hazardous and toxic products. Because nano-structured titanium dioxide also has photocatalytic properties, it is possible to decompose mitoxantrone to carbon dioxide and water by UV radiation.

## Supporting information

S1 File XRD pattern, HRTEM and SEM of anatase, HPLC of mitoxantrone and its degradation products. (DOCX)

S2 File XRD pattern, HRTEM and SEM of anatase, HPLC of mitoxantrone and its degradation products. (PDF)
