# Supplementary material for: Anthracycline antibiotics derivate mitoxantrone—Destructive sorption and photocatalytic degradation
Source: PLoS One. 2018 Mar 13;13(3):e0193116. doi: 10.1371/journal.pone.0193116 (PMC5849306; doi:10.1371/journal.pone.0193116)
Supplement: S2 File — (PDF) [file pone.0193116.s002.pdf]

Supplement Information

Anthracycline Antibiotics Derivate Mitoxantrone - Adsorption and Photocatalytic Degradation

[1]Irena R. Štenglová Netíková*, [1]Luboš Petruželka, [2,3]Martin Šťastný and [1,2]Václav Štengl,

1st Faculty of Medicine, Charles University in Prague, Kateřinská 32, 128 00 Praha 2, Czech Republic

[2]Department of Material Chemistry, Institute of Inorganic Chemistry ASCR v.v.i., 250 68 Husinec-Rez, Czech Republic

[3]Faculty of the Environment, J.E.Purkyně University in Ústí nad Labem, Ústí nad Labem 400 96, Czech Republic

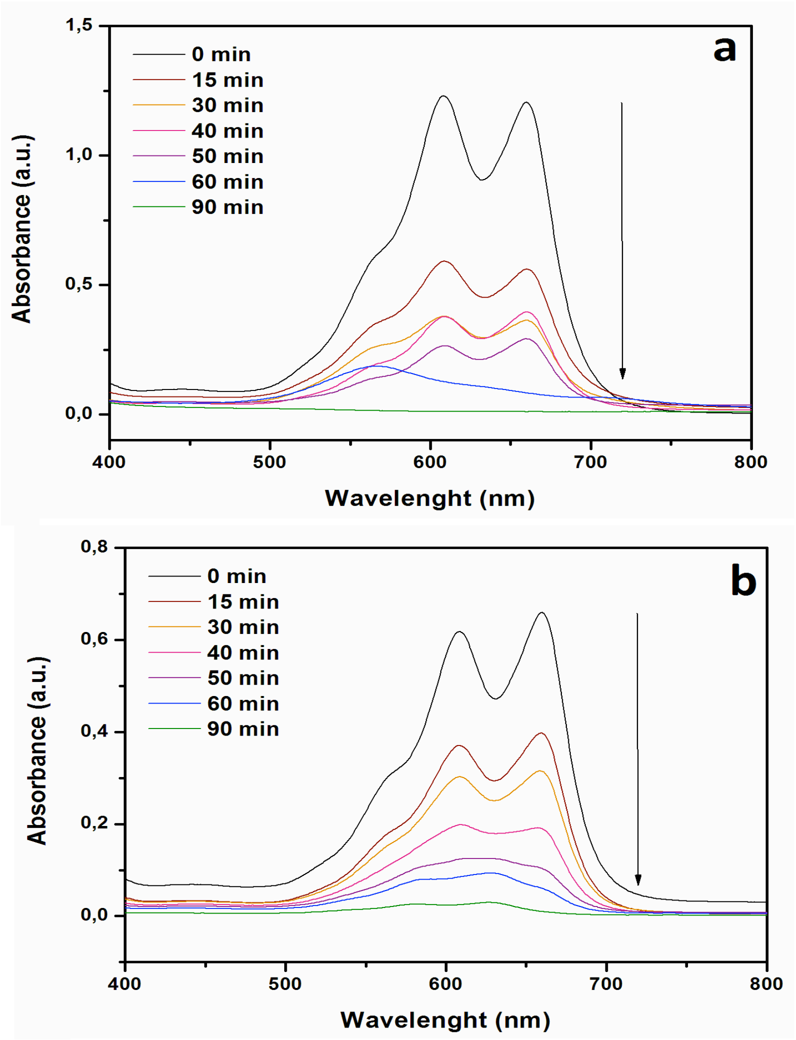

Figure S1. VIS spectra of MTX and its degradation products scanned during the sorption (a) and photocatalytic degradation (b) of MTX on TiO_2 surface

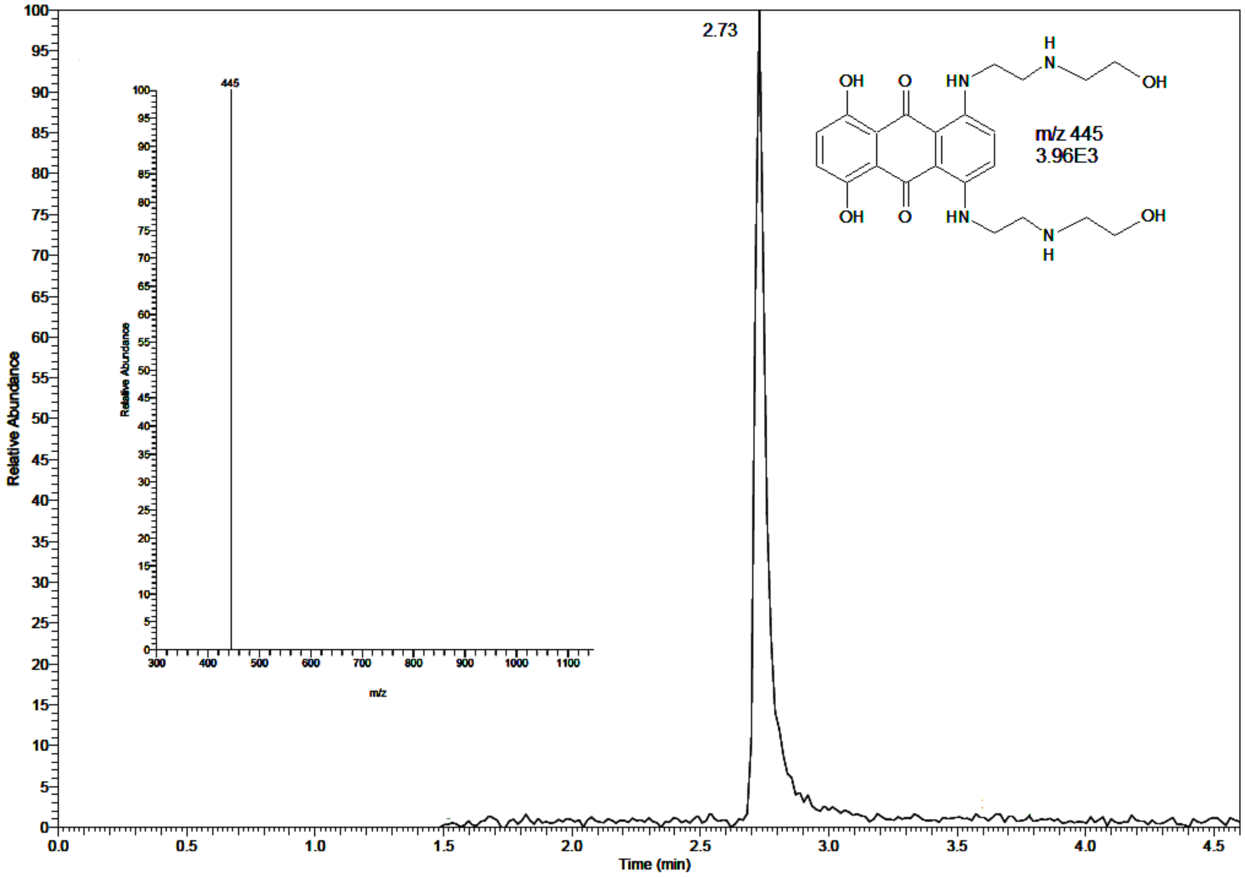

Figure S2. HPLC–MS chromatogram and mass spectra of a standard of MTX.

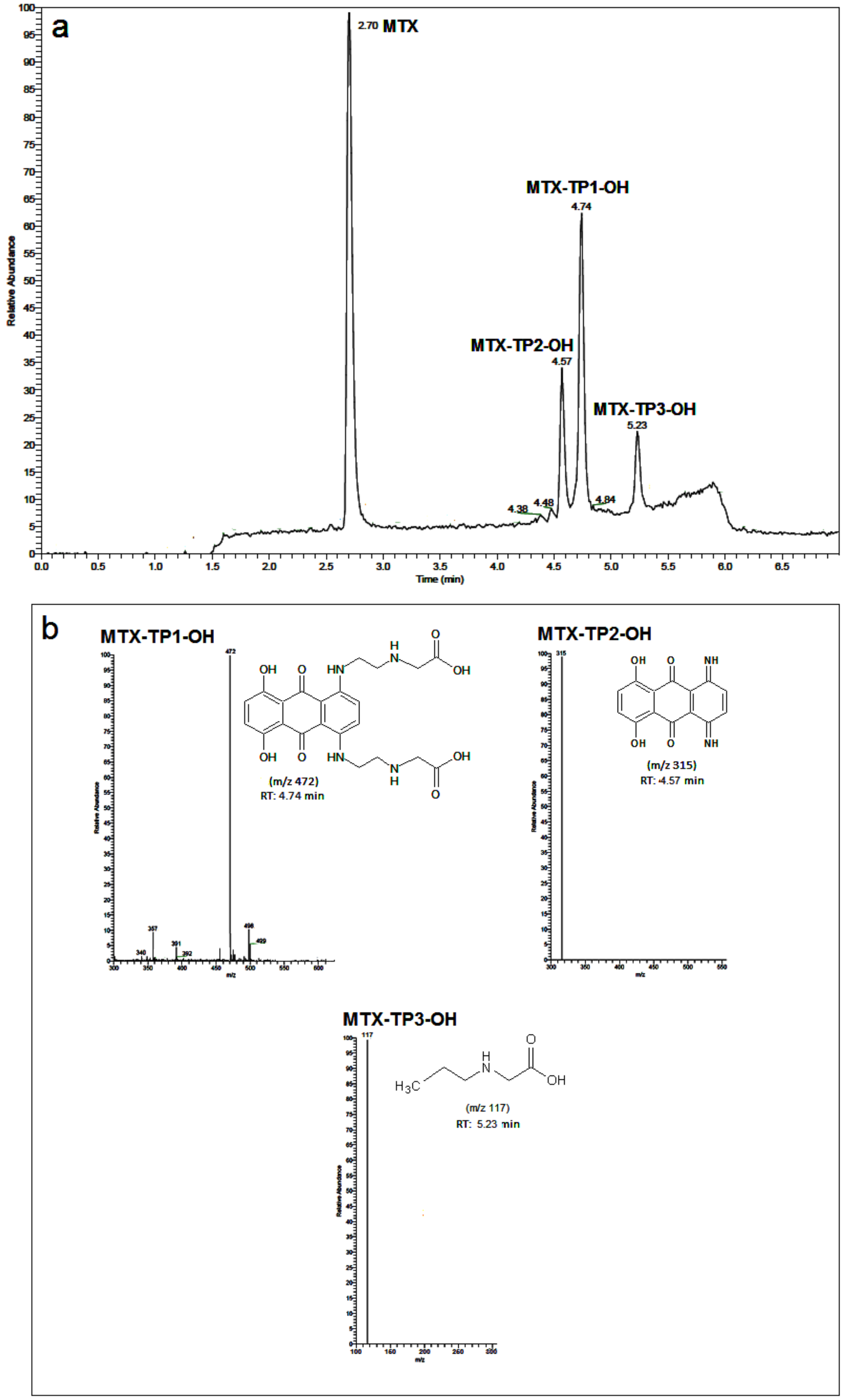

Figure S3. HPLC–MS chromatogram obtained after the reaction with 0.01M NaOH agent solution in pre-determined times (a) and mass spectra of appropriate transformation products of MTX (b).

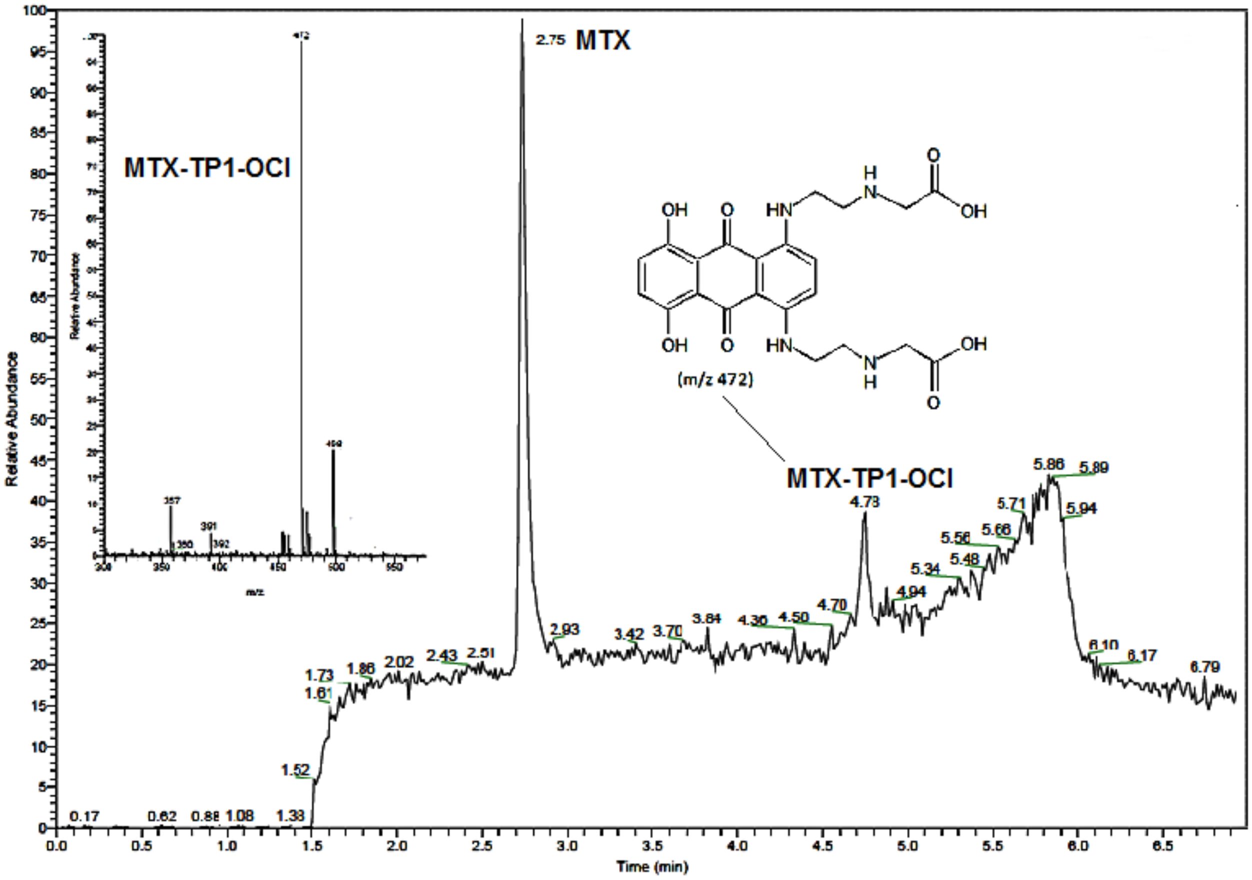

Figure S4. HPLC–MS chromatogram obtained after the reaction with 5% NaOCl agent solution in pre-determined times and mass spectra of appropriate transformation products of MTX

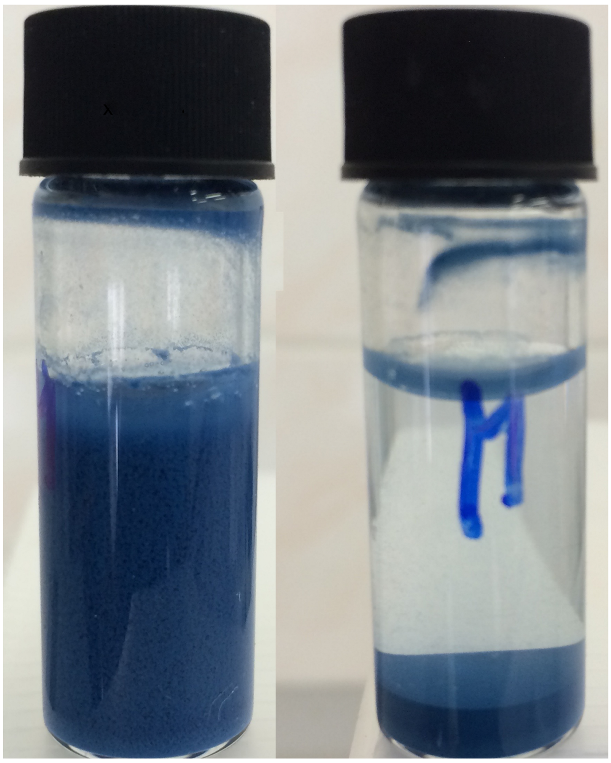

Figure S5. Sorption of 1mL solution of Mitoxantrone (2mg/mL) on the surface of 0.25g TiO_2 within 5 min.

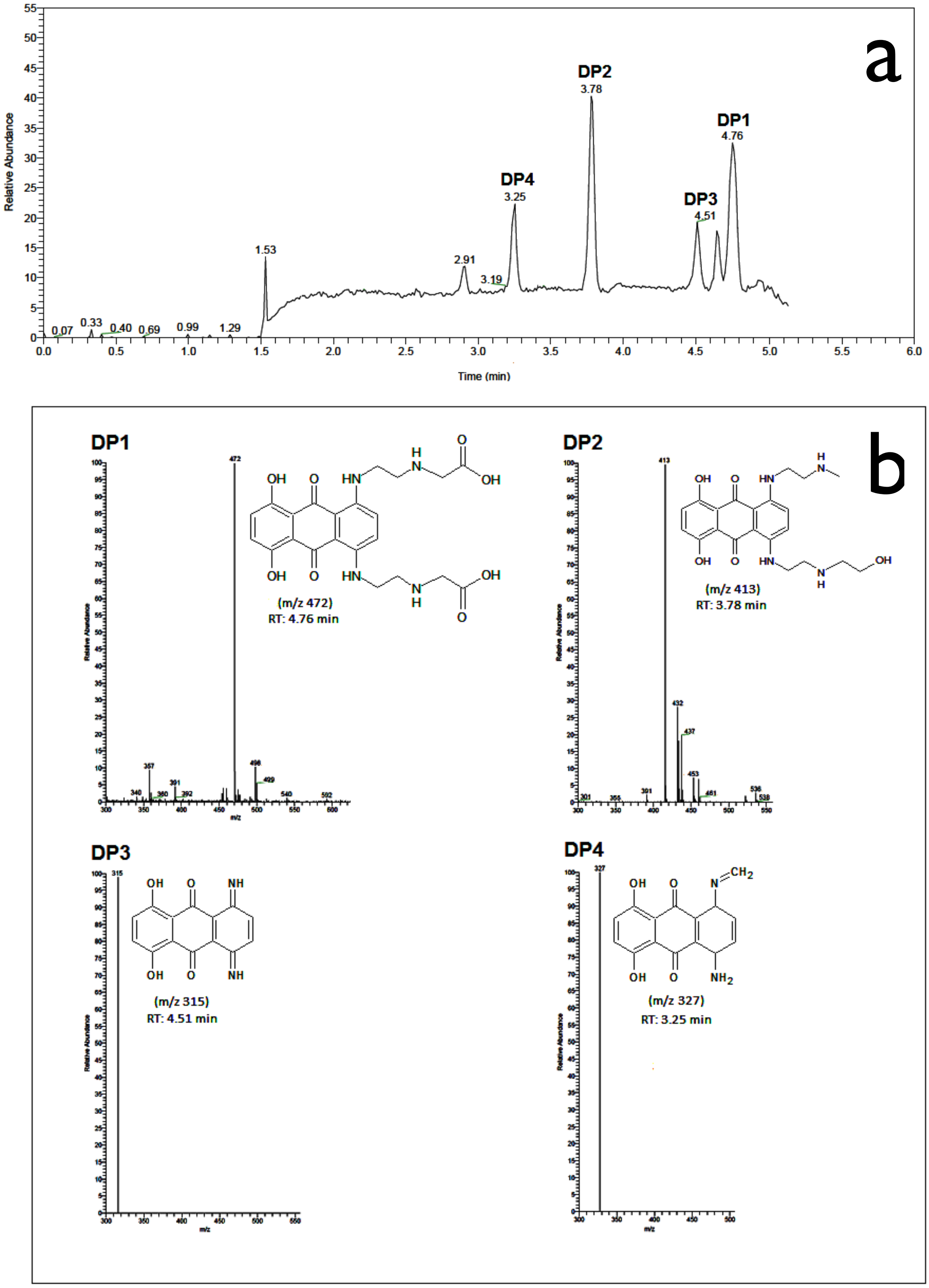

Figure S6. HPLC–MS chromatogram and mass spectra obtained after adsorption in pre-determined times (a) and mass spectra of appropriate degradation products (DPs) of MTX (b).

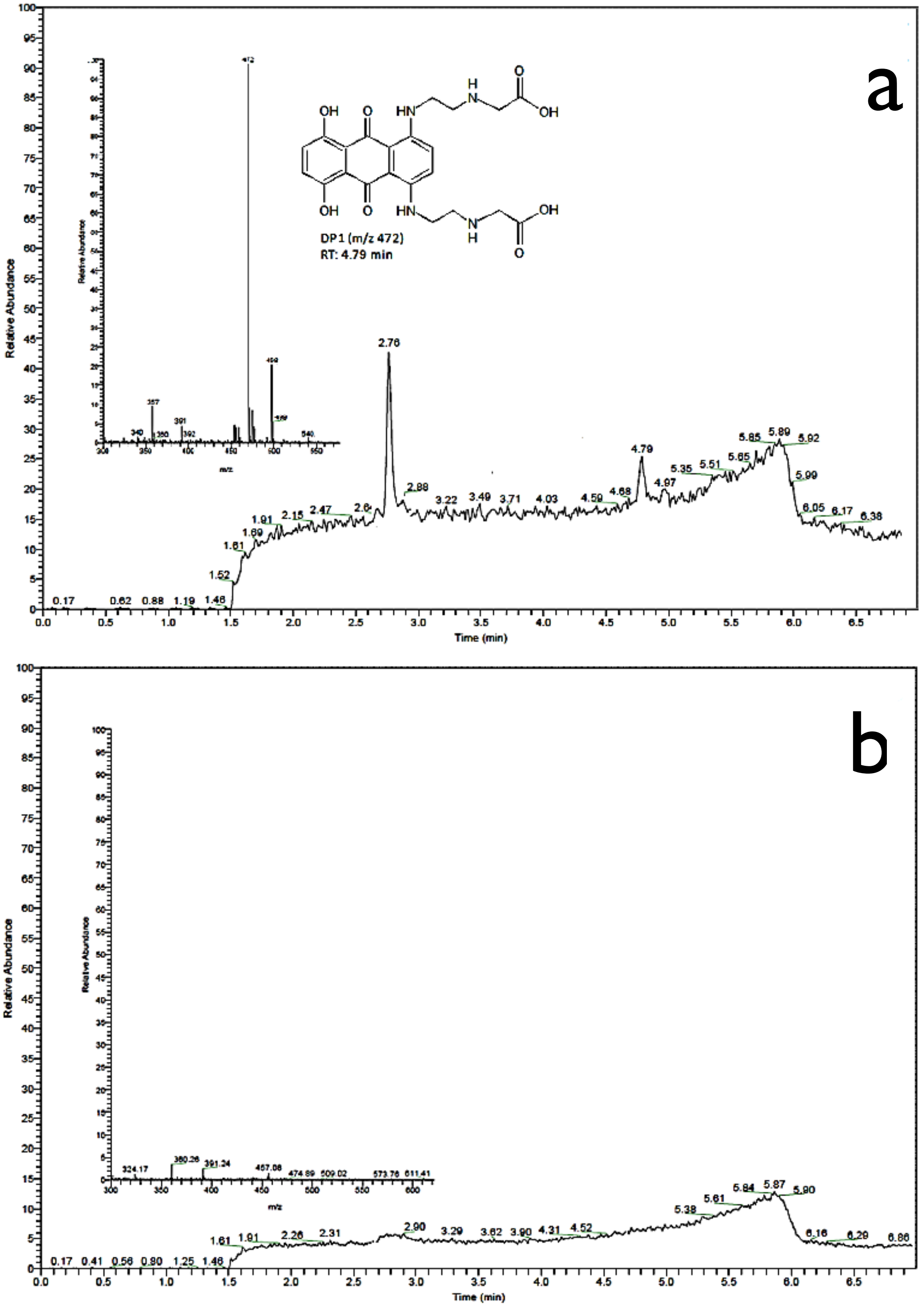

Figure S7. LC–MS chromatogram and mass spectra of photocatalytic degradation in pre-determined times: MTX degradation product 1 (m/z = 472) determined at 15 min (a) and no degradation products after 60 min of the photocatalytic degradation (b).

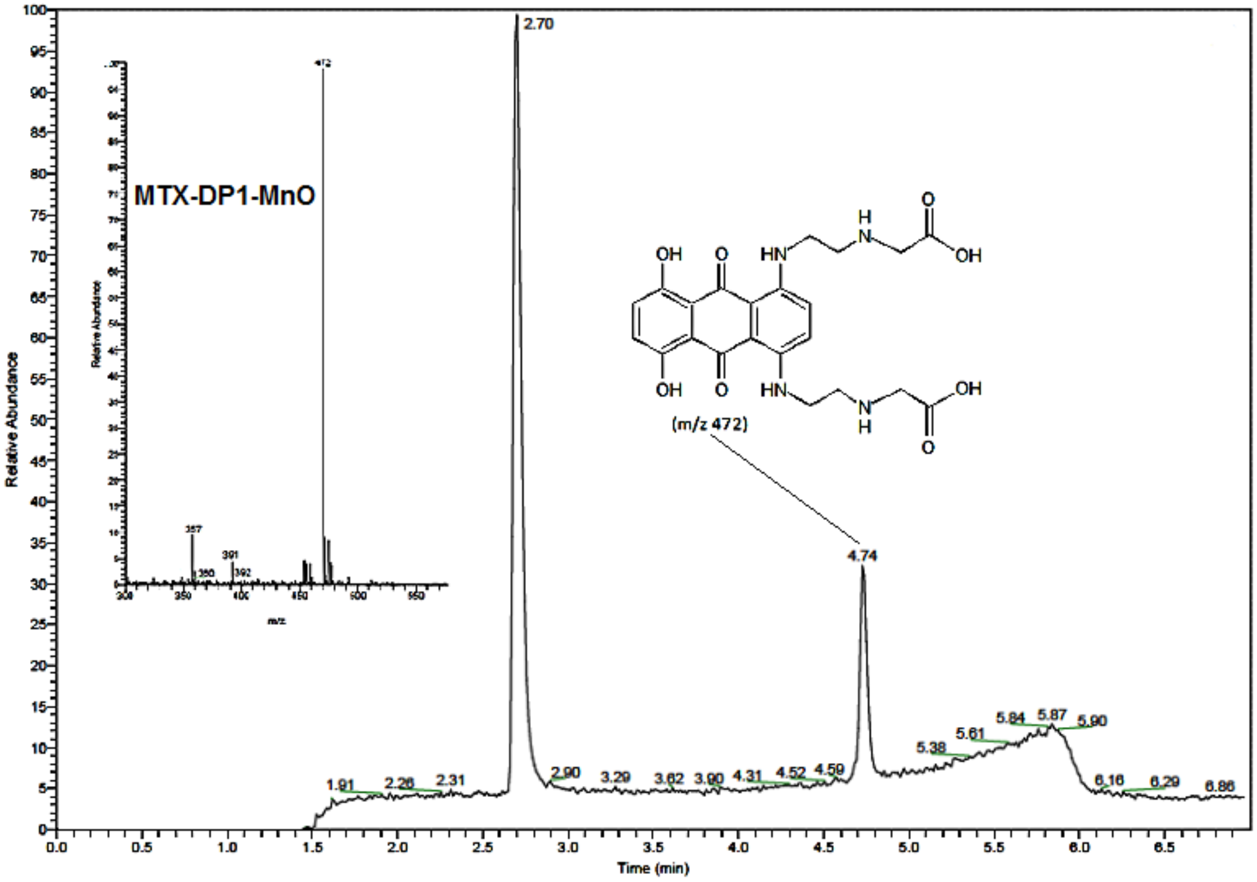

Figure S8. HPLC–MS chromatogram obtained after the using of reactive sorbent MnO2 in pre-determined times and mass spectra of appropriate transformation products of MTX
